# Comparison of the Intestinal Microbiome of Italian Patients with Multiple Sclerosis and Their Household Relatives

**DOI:** 10.3390/life11070620

**Published:** 2021-06-26

**Authors:** Paola Galluzzo, Fanny Claire Capri, Luca Vecchioni, Sabrina Realmuto, Luca Scalisi, Salvatore Cottone, Domenico Nuzzo, Rosa Alduina

**Affiliations:** 1 Dipartimento Scienze e Tecnologie Biologiche, Chimiche e Farmaceutiche, Viale delle Scienze, University of Palermo, 90133 Palermo, Italy; paola.galluzzo@izssicilia.it (P.G.); fannyclaire.capri@unipa.it (F.C.C.); luca.vecchioni@unipa.it (L.V.); 2 Istituto Zooprofilattico Sperimentale della Sicilia “A. Mirri”, Via G. Marinuzzi 3, 90129 Palermo, Italy; 3 Centro Sclerosi Multipla, UOC Neurologia e Stroke Unit, AOOR Villa Sofia Cervello, 90146 Palermo, Italy; sabrina.realmuto@pec.it; 4 Centro Medico di Fisioterapia “Villa Sarina“, Via Porta Palermo 123, 91011 Alcamo, Italy; lucasfrog@libero.it; 5 U.O.C. Neurologia con Stroke Unit A.R.N.A.S. Civico, 90127 Palermo, Italy; salvatore.cottone1@arnascivico.it; 6 Istituto per la Ricerca e l’Innovazione Biomedica, CNR, Via U. La Malfa 153, 90146 Palermo, Italy

**Keywords:** multiple sclerosis, microbiome, Ruminococcaceae, Desulfovibrionaceae Christensenellaceae, Clostridiales, Bacteroidaceae, Tannerellaceae, Veillonellaceae, Burkholderiaceae

## Abstract

Multiple sclerosis (MS) is a chronic immune-mediated disease of the central nervous system, caused by a combination of genetic and environmental factors. In recent years, a role in MS pathogenesis was assigned to the gut microbiota. However, different signatures of gut dysbiosis have been shown to depend on environmental factors, like diet and lifestyle. In this study, we compared the gut microbiome in MS patients and their household healthy relatives sharing lifestyle and environmental factors. Faecal metagenomic DNA was extracted and the V3–V4 regions of the conserved bacterial 16S ribosomal RNA gene were amplified and sequenced. While overall bacterial communities were similar, specific families differed between healthy and MS subjects. We observed an increase in Ruminococcaceae, Christensenellaceae, Desulfovibrionaceae, Clostridiales, and Family XIII in MS patients, while Bacteroidaceae, Tannerellaceae, Veillonellaceae, and Burkholderiaceae were more abundant in healthy controls. In addition, principle coordinate analysis showed that the gut microbiome of all MS patients formed a cluster being less diverse than the household relatives and that gut microbiota of MS patients with EDSS 4.5–7 formed a distinct cluster in respect to their controls. Overall, our study is consistent with the hypothesis that MS patients have gut microbial dysbiosis and evidenced the importance of environmental factors in shaping the gut microbiome.

## 1. Introduction

Multiple sclerosis (MS) is a chronic demyelinating inflammatory disease of the central nervous system (CNS). In the CNS of MS patients, multiple demyelinating lesions induce various neurological symptoms. Most MS patients (~85%) have relapsing-remitting MS (RRMS) characterized by new or increasing neurologic symptoms followed by periods of partial or complete recovery (remissions). Disease activity in RRMS is correlated with periodic activation of myelin-specific T cells, but the mechanisms that regulate the aggressiveness of these self-reactive T cells are still largely unknown [1,2,3,4]. However, some RRMS patients proceed to a progressive phase (PPMS) characterized by the accumulation of irreversible neurological disabilities in later disease stages [5,6]. Several studies carried out on mouse models of MS, such as experimental autoimmune encephalomyelitis (EAE), demonstrated the central contribution of CD4+ T lymphocytes in the pathogenesis [7,8]. In particular, CD4+ T helper 17 lymphocytes are involved in the production of different interleukins that contribute to tissue damage during chronic inflammation; and, IL-17, the main secretion product of Th17 cells, is responsible for maintaining a pro-inflammatory environment and is increased in EAE mice and MS pathophysiology [8,9,10].

The causes leading to MS onset are complex and not entirely understood. Epidemiological studies have revealed that both genetic and environmental factors are involved in the disease development including deficiency of vitamin D, obesity in early life, infection with Epstein–Barr virus, cigarette smoking, and salt intake [11]. Although genetics can contribute to susceptibility to multiple sclerosis, almost two-thirds of monozygotic twins are discordant, suggesting a major role of the environment. Recent evidence considered the altered gut microbiota as one of the major environmental factors contributing to MS susceptibility or protection [12].

The gut microbiota represents the collection of microorganisms (bacteria, archaea and eukarya) colonising the gastrointestinal (GI) tract and has been estimated to contain ∼10^13^–10^14^ microbial cells [13,14,15,16]. The gut microbiome is the genome of all the microorganisms, symbiotic and pathogenic, inhabiting the gut, accessible through high throughput sequencing technology. In recent years, numerous studies have shown that bacteria and their metabolites have a key role in health and are essential to various host physiologic functions, such as immunoregulation, pathogen prevention, energy harvest, metabolism, development, and homeostasis of the nervous system [17,18,19]. Until today, how gut microbial imbalance (dysbiosis) may lead to dysfunction of host machinery, thereby contributing to pathogenesis and/or progression toward a broad spectrum of diseases, is unclear.

Generally, MS patients are characterized by gut dysbiosis in respect to healthy controls [9,20,21,22,23,24,25,26,27,28]. Firmicutes, such as Streptococcaceae, Ruminococcaceae, and Lachnospiraceae, which can induce inflammatory effects, were increased in MS patients in some studies [9,21,23,25,26,27,28] and decreased in other ones [20,21,25,26,27]. On the other hand, Bacteroidetes, Prevotellaceae and Bacteroidaceae [9,20,21,22,27,28] were frequently reduced in MS patients and some of them, i.e., *Prevotella*, are related to increased intestinal Th17 cell frequency [9,24]. Proteobacteria, such as Desulfovibrionaceae [23], and Actinobacteria, such as Bifidobacteriaceae [21,27], were more abundant in MS patients.

Despite these important milestones, the role of the microbiota on the host and vice versa has been poorly understood. This could be due to several technical reasons, such as the lack of standardized study protocols, different sampling methods, or diverse ribosomal gene sequencing approaches. Other confounders could be the environmental factors, such as diet and external temperatures, that are known for modulating the gut microbiota [29,30].

In this work, the gut microbiome of Italian patients with multiple sclerosis and their relatives living in the same house and sharing similar eating habits and lifestyles was investigated to reduce the effects due to environmental factors, like diet, climate, or geographical place.

## 2. Materials and Methods

### 2.1. Study Population

**Figure 1:**
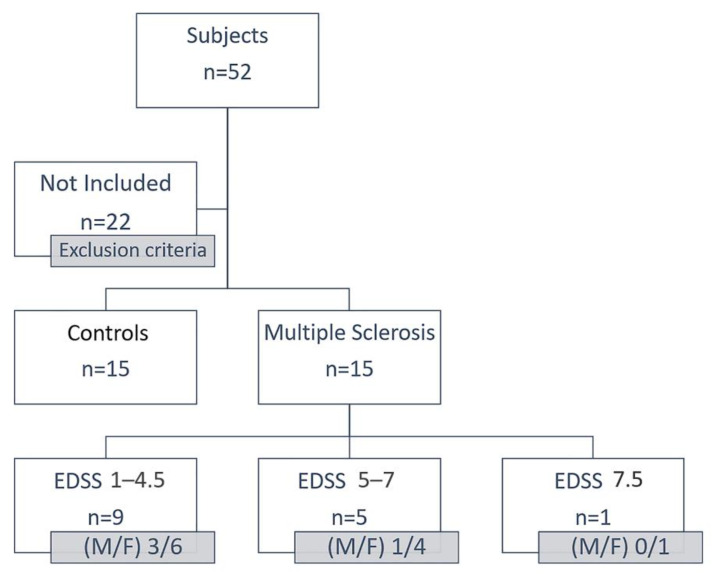
Scheme of recruitment of the subjects of this study. Thirty subjects were recruited: n = 15 controls and n = 15 MS patients. These patients were classified according to the EDSS value into three groups: EDSS 1–4.5 (9 patients), EDSS 5–7 (5 patients), EDSS 7.5 (1 patient).

In this study, 52 subjects were recruited. Exclusion criteria included the diagnosis of a significant neurological disease other than MS, the use of antibiotics or anti-inflammatory drugs in the last 6 months, excessive alcohol intake or drug abuse, and investigator uncertainty regarding the willingness or ability of the patient to understanding the protocol of faecal sample collection (Figure 1).

Participants were recruited from 2 MS centres: Alcamo (TP) and Palermo (PA) of Sicily. To reduce the dietary variability, a household relative (wife/husband/daughter) for each MS patient was recruited. All the participants were requested to follow the same diet in the two weeks before the faecal collection. The patients and the controls coming from the same town were subjected to the same climatic and environmental conditions (temperature, distance from the sea, smog, pollution). The Ethics Committees of each participating centre reviewed and approved the protocol. The study was conducted following the Declaration of Helsinki. The Ethics Committees number for this study is 7/2019. Fifteen MS patients, aged 28–66 years and with an Expanded Disability Status Scale (EDSS) between 0 and 7, were included together with their relative controls. The data of the MS patients and the controls are summarized in Table 1.

### 2.2. Sample Collection, Genomic DNA Extraction, PCR Amplification and Sequencing

Faecal samples were collected in tubes containing RNA Later and maintained at 4 °C within 24 h of receipt. Metagenomic DNA was extracted using the QIAmp DNA Stool Mini Kit (Qiagen, West Sussex, UK), following the manufacturer’s instructions. After quantification by NanoDrop 2000c spectrophotometer (Thermo Fisher Scientific, MA, USA), DNA was diluted to obtain a concentration of 50 ng/µL and used as a template to amplify the region V3-V4 of the 16S rDNA gene using primers described previously [31]. Amplification products were sequenced in one 300-bp paired-end run on an Illumina MiSeq platform (BMR Genomics, Padova, Italy).

### 2.3. Raw Data Processing

The raw 16S rDNA data were processed using QIIME2 environment [32] (https://qiime2.org, accessed on 1 November 2020) as paired-end sequences. DADA2 plug-in was used to filter, remove chimaeras and denoise all produced sequences to obtain the Operational Taxonomic Units (OTUs). After removing singletons, the taxonomy of each OTU was determined, with a 97% similarity level, through the implementation of the SINA classifier (using the SILVA dataset available at https://www.arb-silva.de/ngs/, accessed on 1 November 2020) [33]. Unclassified OTUs were not assigned. Rarefaction analysis was carried out plotting the number of the observed OTUs against the total number of filtered reads for each sample. Sequences were deposited in GenBank (BioProject ID: PRJNA684124, December 2020).

For each sample, the number of observed OTUs and the percentages of relative abundances of phyla, orders, classes, and families were determined. Similarities among the studied samples were observed using principal coordinate analysis (PCoA) through the implementation of software package PRIMER 6 (November 2020) [34]. The analysis was based on the Bray–Curtis distance matrix. Furthermore, METAGENassist (November 2020) [35] was used to distinguish the microbial species based on their metabolic activity.

Alpha diversity, Abundance-based Coverage Estimator (ACE), Chao1, Shannon-Wiener diversity, H’, and Simpson index, 1-D, and evenness, e (equitability assumes a value between 0 and 1 with 1 being complete evenness), were estimated to determine the specific faecal microbial richness and diversity. Good’s coverage was estimated to evaluate the completeness of sampling.

## 3. Results

### 3.1. Bacterial Communities’ Diversity

Microbiome analysis of faecal samples of 15 MS patients and 15 controls (Table 2) showed that MS samples (here after indicated with number-S) contained between 105 and 283 OTUs for a total of 5510 corresponding to 13 bacterial phyla, 20 classes, 39 orders and 62 families. Control samples (indicated with number-C) contained between 82 and 384 OTUs for a total of 5600, which allowed us to identify 15 phyla, 25 classes, 54 orders and 87 families. Good’s coverage, used to estimate the completeness of sampling, showed a high level (0.99–1.00) in the identification of bacterial groups.

The analysis of various diversity indices, i.e., the abundance-based richness estimators (Chao1 and ACE) did not reveal significant differences between the two groups, indicating good diversity. Bacterial diversity estimated by the Shannon–Wiener index varied from 2.13 to 2.80 in S samples, and 2.03 to 3.13 in C samples, indicating similar diversity values between the two studied groups. Simpson index (0.09–0.21 in S, 0.07–0.20 in C) and evenness (0.65–0.81 in S, 0.68–0.85 in C) revealed no significant difference between MS and control subjects (Table 2).

### 3.2. Faecal Bacterial Communities’ Taxonomic Composition

**Figure 2:**
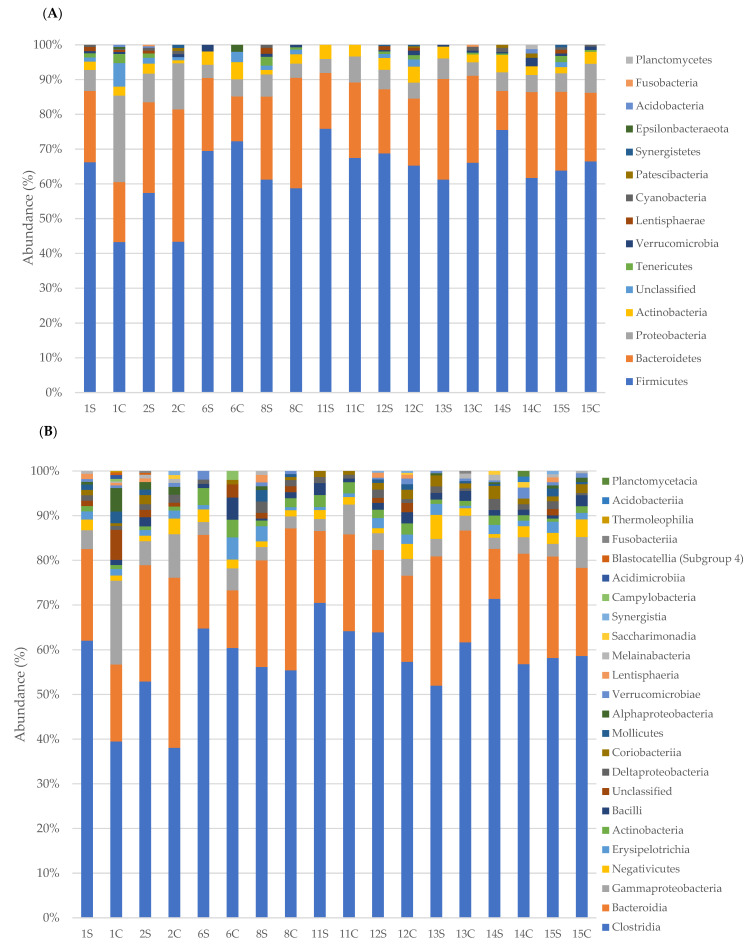

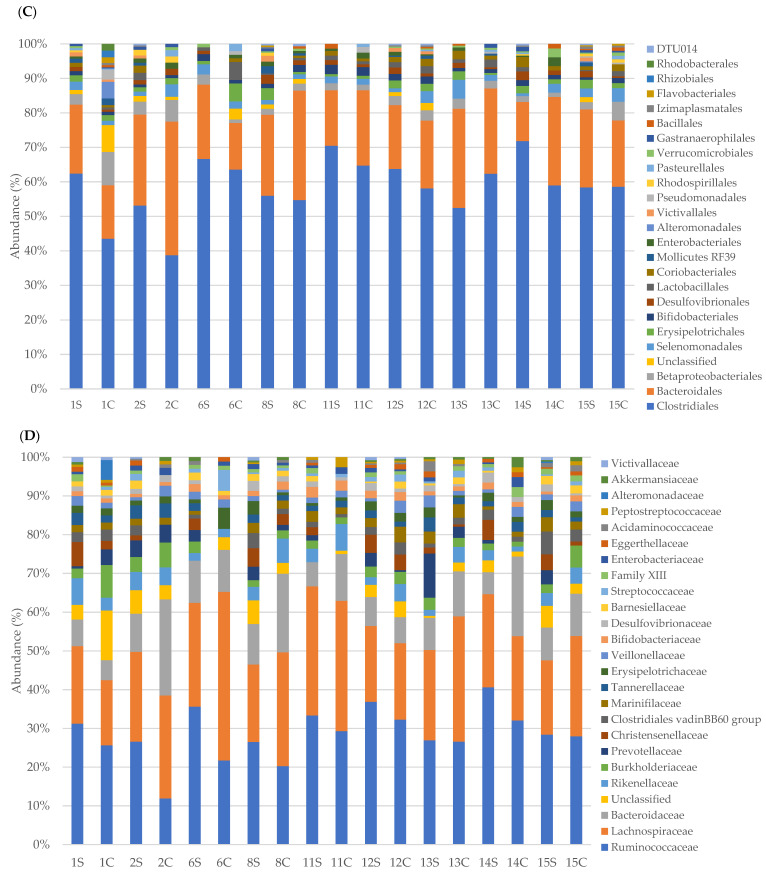
Relative abundance (%) of faecal bacterial communities in MS patients with EDSS 1–4.5 and controls. Bacterial communities were studied at the phyla (**A**), classes (**B**), orders (**C**), and families (**D**) levels.

**Figure 3:**
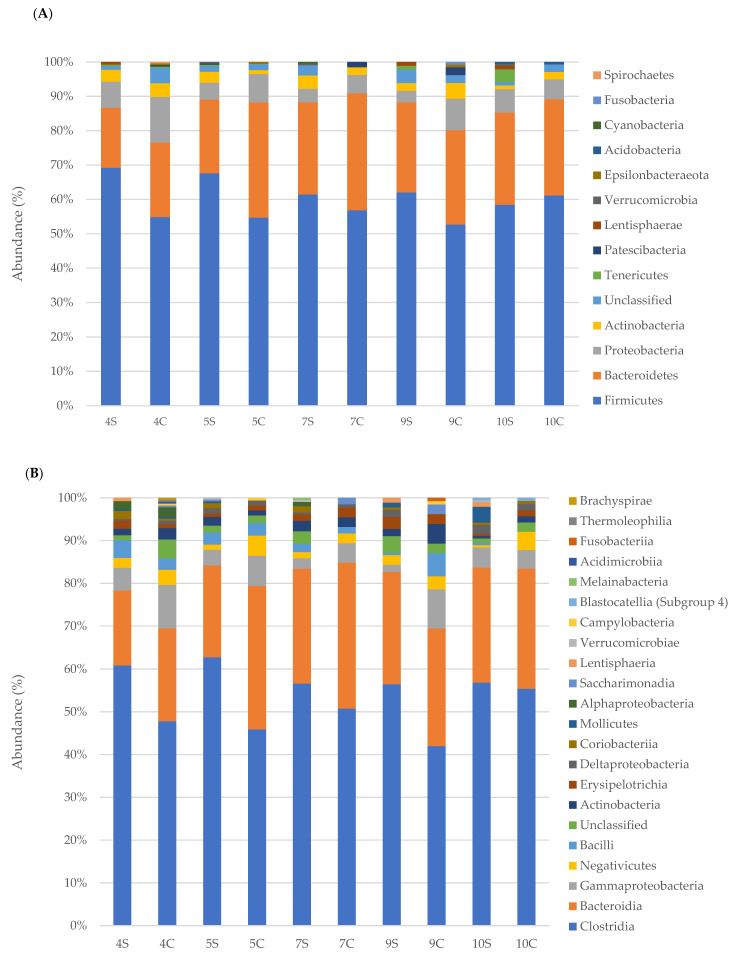

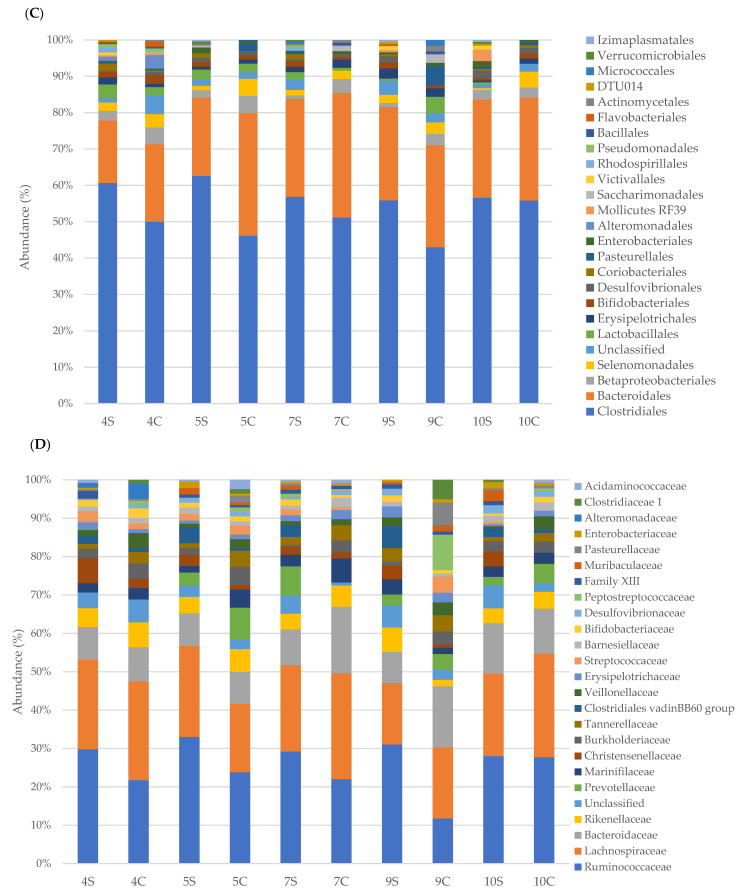
Relative abundance (%) of faecal bacterial communities in MS patients with EDSS 5–7 and controls. Bacterial communities were studied at the phyla (**A**), classes (**B**), orders (**C**), and families (**D**) levels.

Taxonomic analysis was performed for phyla, orders, classes and families and the results are presented on the basis of the EDSS (1–4.5 and 5–7) of MS patients compared to the household relatives (Figure 2 and Figure 3). Since only a PPMS patient was recruited, it was excluded from the study.

In particular, taxonomic classification revealed that the most dominant phylum in faecal samples of MS patients was Firmicutes with an average relative abundance of 65.6% ± 6 followed by Bacteroidetes (22% ± 5), Proteobacteria (5.5% ± 1.4), and Actinobacteria (3% ± 1.1). Data from control samples revealed a lower mean abundance of Firmicutes (59% ± 8.7) and a higher mean percentage of Bacteroidetes (25.3% ± 7) and Proteobacteria (8.4% ± 5.6). Regarding Actinobacteria, no significant difference (3% ± 1.2 in controls) was registered. Minor phyla, such as Tenericutes and Lentisphaerae, were more present in MS patients (0.9% ± 1.09 and 0.6% ± 0.61, respectively) than control subjects (0.43% ± 0.7 and 0.1% ± 0.26, respectively).

**Figure 4:**
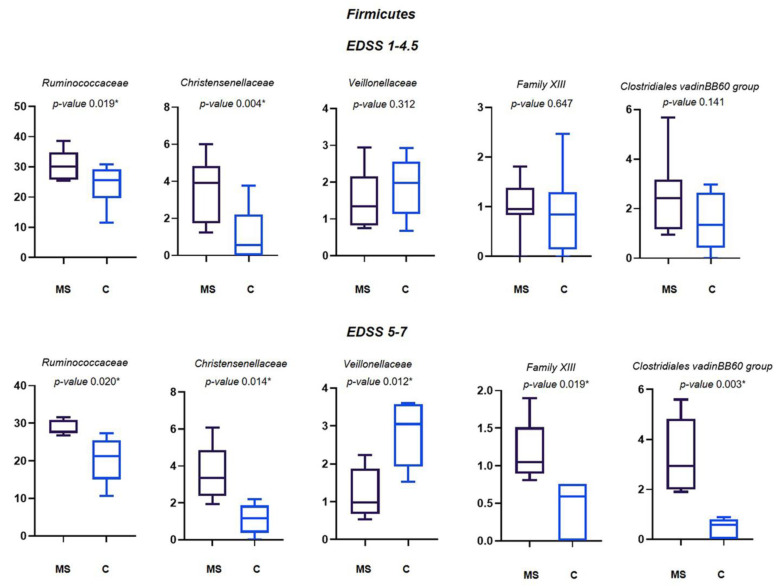
Relative abundance (%) of the main families of Firmicutes differently abundant in MS patients and their controls. Means ± SEM of the relative abundance of Ruminococcaceae, Christensenellaceae, Veillonellaceae, Family XIII and Clostridiales vadin BB60 group families, detected on faecal samples of MS patients with EDSS 1–4.5 and EDSS 5–7, and their relatives. The central line indicates the median value. Families with statistically significant differences (*p* ≤ 0.05) are indicated with *.

**Figure 5:**
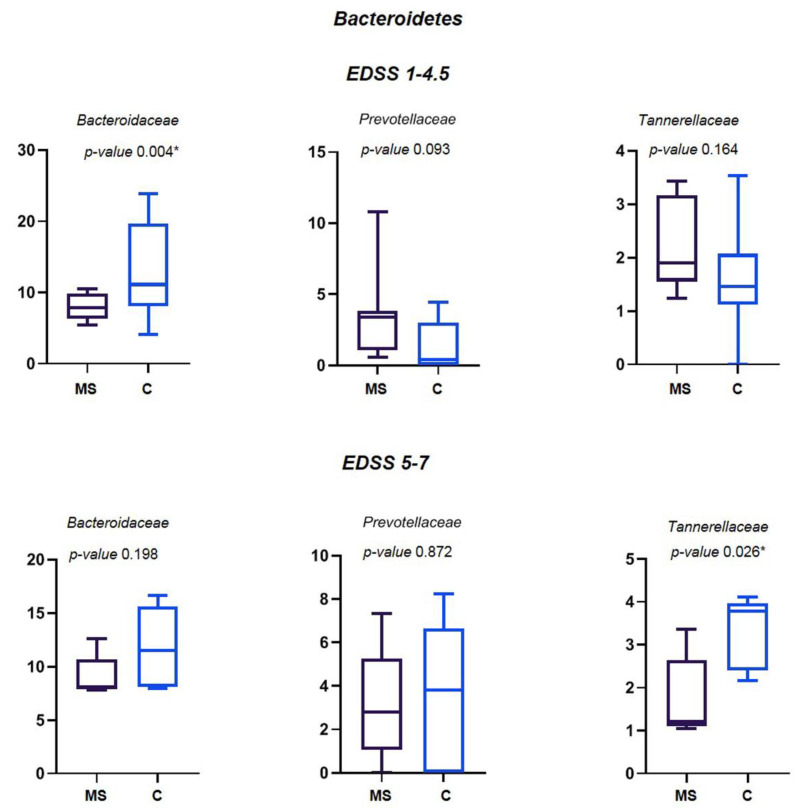
Relative abundance (%) of the main families of Bacteroidetes differently abundant in MS patients and their controls. Means ± SEM of the relative abundance of Bacteroidaceae, Prevotellaceae and Tannerellaceae families, detected on faecal samples of MS patients with EDSS 1-–4.5 and EDSS 5–7, and their relatives. The central line indicates the median value. Families with statistically significant differences (*p* ≤ 0.05) are indicated with *.

**Figure 6:**
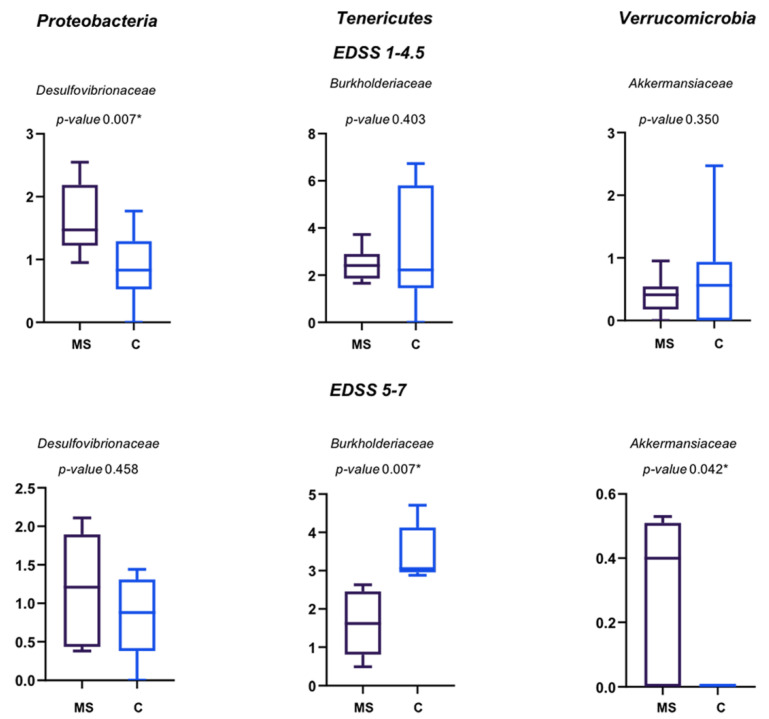
Relative abundance (%) of the main families of Proteobacteria, Tenericutes and Verrucomicrobia differently abundant in MS patients and their controls. Means ± SEM of the relative abundance of Desulfovibrionaceae, Burkholderiaceae and Akkermansiaceae families, detected on faecal samples of MS patients with EDSS 1–4.5 and EDSS 5–7, and their relative controls. The central line indicates the median value. Families with statistically significant differences (*p* ≤ 0.05) are indicated with *.

Taxonomic classification of bacterial families revealed that the most dominant family in faecal samples of MS patients was Ruminococcaceae (30% ± 4), followed by Lachnospiraceae (22% ± 3.8), Bacteroidaceae (8.3% ± 1.9) and Rikenellaceae (3.6% ± 21.5). Control subjects revealed a lower mean abundance of Ruminococcaceae (22.5% ± 6.2) and a higher mean percentage of Lachnospiraceae (24.7% ± 7), Bacteroidaceae (12.5% ± 5.6) and Rikenellaceae (4.1% ± 1.7). Bacteria belonging to other families (Christensenellaceae, Clostridiales, Prevotellaceae and so on) were minor components and were not found in all samples. The relative abundance of specific bacterial families in MS patients compared to the healthy relatives is reported in Figure 4, Figure 5 and Figure 6 according to EDSS values (Tables S1 and S2).

Among Firmicutes, Ruminococcaceae, Christensenellaceae, Veillonellaceae, Family XIII and Clostridiales vadin BB60 group families showed statistically significant differences among MS patients and their controls. Specifically, all but Veillonellaceae, increased in MS patients. Ruminococcaceae and Christensenellaceae were increased in the MS patients with both the EDSS of 1–4.5 and 5–7 in respect to their controls. The differences of mean average of Veillonellaceae and Family XIII were significant only for EDSS 5–7 (Figure 4).

Among Bacteroidetes, a statistically significant decreased abundance of Bacteroidaceae was found for MS patients with EDSS 1–4.5 and of Tannerellaceae for MS patients with EDSS 5–7 (Figure 5). Prevotellaceae, found decreased in other studies [9], did not change in this study.

A significant different abundance of Desulfovibrionaceae was found for MS patients with EDSS 1–4.5, while Burkholderiaceae and Akkermansiaceae were significantly different in abundance for MS patients with EDSS 5–7; Burkholderiaceae were decreased while Desulfovibrionaceae and Akkermansiaceae increased in MS patients (Figure 6).

**Figure 7:**
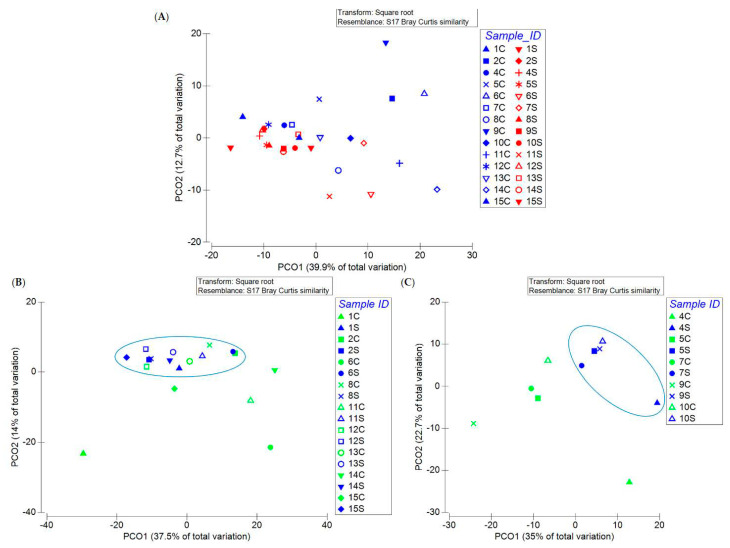
Principle coordinate analysis (PCoA) plot of all the MS patients (**A**), of MS patients with EDSS 1–4.5 (**B**) and of MS patients with EDSS 5–7 (**C**) and their relative controls. Circles indicate the samples forming a cluster.

As written above, the samples did not display different microbial diversity. Interestingly, when the MS patients and the controls were analysed, the PCoA plot showed that 11 out of the 14 samples collected from MS patients had a more similar microbial composition than the healthy controls (6 out of the 14) (Figure 7A). Following this comprehensive analysis, we applied PCoA to the gut microbiome of MS patients with EDSS 1–4.5 and 5–7 and that of their household relatives. Interestingly, we found that all of the MS samples with EDSS 1–4.5 clustered and that most of the controls fell in the same cluster (Figure 7B), while MS samples with EDSS 5–7 formed a distinct cluster from their controls (Figure 7C).

### 3.3. Phenotypic and Metabolic Inference

The microbial metabolic profile was analyzed by METAGENassist. All samples contained bacteria with the metabolic potential to oxidize ammonia, to degrade cellulose and xylan, to reduce sulfide and nitrite, and to dehalogenate and fix nitrogen. Conversely, a few samples contained bacteria with atrazine metabolism (1S, 1C, 3S, 4S, 4C, 11C, 13C), able to degrade chitin (5S, 6S, 6C, 7S, 7C), denitrify, degrade lignin and oxidize sulfur (1S, 1C) (Figures S1 and S2). Bacteria able to oxidize ammonia, degrade cellulose and xylan, and dehalogenate were more present in controls than MS subjects. Conversely, bacteria able to reduce sulfates and oxidize sulfides were more present in MS patients.

## 4. Discussion

In this study, we evaluated, for the first time, the gut microbial composition of a cohort of samples coming from the same location in Sicily (Island of Southern Italy), sharing lifestyle and environmental factors; besides, the gut microbiome of healthy people (average years 21–69) was compared with that of relatives affected by multiple sclerosis.

Studies on the microbiome of individuals living in the same house can represent a valid strategy to mitigate the differences due to the heterogeneity of otherwise unrelated controls and it was already demonstrated that matched household controls share similar gut, skin, and oral microbiomes [36,37,38,39]. To the best of our knowledge, only in a few studies, samples for microbiome analysis were collected from controls from the MS patient’s house [24,40].

According to the latest survey carried out in 2020, MS affects 126,000 people in Italy (an estimated 198 cases per 100,000 inhabitants), with a rate twice as high in women compared to men. In Europe, Italy is identified as one of the high-risk areas for MS. Overall 85% of cases are reported in Northern Italy, with a higher prevalence in Lombardy (16%), twice as high as in Sicily (8%). The national mean of MS cases is 5% (www.aism.it, accessed on 1 February 2021).

Alterations of the gut microbiota are associated with various neurological and psychiatric diseases, including Parkinson’s disease, Alzheimer’s disease, major depressive disorder, autism and MS [41,42].

As far as we know, only one study has already investigated the microbiome of Italian MS patients with an EDSS between 1 and 5, coming from Northern Italy [9]. In this latter case, the duodenal mucosal microbiome of MS patients was investigated, while we analysed faecal samples [9]. Although the four main phyla were common, the percentage of the main phyla detected was discordant between our study and the study carried out in Northern Italy [9].

Indeed, our data showed an increase of Firmicutes (59% vs. 52%), Bacteroidetes (25% vs. 10%) and Actinobacteria (3% vs. 1.4%) and a decrease of Proteobacteria (8% vs. 18%) in healthy people. Even if we cannot rule out that these differences could be due to the different sampling origin (faecal vs. duodenal mucosa), we could surmise that sun exposure, eating habits and other environmental factors could influence the microbial composition of intestinal samples of people from Northern and Southern Italy. Starting from these differences, the main intestinal dysbiosis of the Northern Italy study is due to the increase of Bacteroidetes and Proteobacteria and the decrease of Firmicutes in MS patients; differently, in our samples, we found the opposite trend with the increase of Firmicutes and the decrease of Bacteroidetes and Proteobacteria. These large differences show that the environment and the sampling are two key factors in the analysis of the gut microbiome. The Firmicutes/Bacteroidetes ratio was quite similar among MS patients (those with EDSS 1–4.5 having 3.2 and those with EDSS 5–7 having 2.6) and healthy controls having 2.4. In contrast, in the Northern Italy samples, healthy controls displayed an increased Firmicutes/Bacteroidetes ratio (5.2), while MS patients showed a ratio similar to the MS patients of this study (2.4).

Moreover, our study was carried out on healthy people living in the same house as the MS patient, to reduce the impact of environmental factors.

Controls showed a significant increase in Bacteroidaceae, Tannerellaceae, Veillonellaceae and Burkholderiaceae families compared to MS patients. However, differences between household relatives and MS patients varied according to EDSS: the number of bacterial families with significant differences between MS patients and controls is smaller in the group of patients with EDSS 1–4.5 than in patients with higher EDSS. This may show that the degree of dysbiosis increases as the disease progresses.

Ruminococcaceae and Christensenellaceae are the two families whose abundance is higher in MS patients with EDSS 1–4.5 and 5–7 than in household controls. The Ruminococcaceae family is correlated with a pro-inflammatory situation and an increased presence of pro-inflammatory mediators, such as TNFα, IL-6, and IL-17, and is associated with vitamin D reduction [43], which can be linked to the very low levels of the vitamin reported by most patients (below 30 ng/mL, personal communication). Gut dysbiosis leads to increased permeability of the gut barrier and blood–brain barrier (BBB) with implications for systemic and CNS autoimmunity [44]. Although it was excluded from the study, analysis of the gut microbiome of the only MS patient with EDSS 7.5 showed a significant decrease of Ruminococcaceae compared to the household control (9.3% vs. 32.7%, data not shown). This unexpected result points to the importance of carrying out further studies to better understand the involvement of this bacterial family in MS.

The Christensenellaceae family has been identified as one of the most heritable taxa, therefore its presence in the gut microbiome is influenced by the genetic composition of the host [38]. The increase of Ruminococcaceae and Christensenellaceae in MS patients was observed in other independent studies [23,25,26]. Furthermore, an increase of Christensenellaceae has been associated with Parkinson’s disease [41], metabolic disorders and gastrointestinal diseases, as well as a potential indicator of mortality risk in patients with neurocritical disease [42].

Clostridiales are more abundant in MS patients than in healthy controls, like reported elsewhere [21,26,28]. Clostridiales can produce toxins, i.e., the Epsilon toxin, that could cross the blood–brain barrier and induce demyelination processes related to the symptoms of MS [45]. Contrarily, the Bacteroidaceae family is assigned an anti-inflammatory role and it was found to be more abundant in household controls, in agreement with the results of another study carried out in Japan [21]. As far as we know, Family XIII, Desulfovibrionaceae, Tannerellaceae, and Burkholderiaceae have been never found to be differently abundant in the gut microbiome of MS patients. Although the Prevotellaceae family was reported to have a role in limiting Th17 lymphocyte intestinal expansion [9], our findings did not show any correlation between the MS gut microbiome and this bacterial family.

Although no bacterial biodiversity was evidenced by the biodiversity indices, the PCoA analysis showed a cluster of microbiome samples of MS patients, mainly with EDSS 4.5–7, regardless of age and gender.

The bacteria present in all faecal samples are ammonia oxidizers, cellulose degraders and capable of dehalogenation, in particular, they seem more abundant in samples of control subjects. Sulphate reducing bacteria, such as Desulfovibrionaceae, seem more abundant in faecal samples of patients with MS. These bacteria were found in human faeces and were related to inflammatory bowel diseases [46].

Studies of correlation among MS and environmental factors in different geographical areas can help us to understand how the environmental and climatic factors can impact the prevalence of MS. The identification and the characterization of microbes or microbial consortia responsible for gut dysbiosis in MS patients is of key importance to prepare interventional strategies in the future that modulate the gut microbiota in a rational and evidence-based manner.

## 5. Conclusions

**Figure 8:**
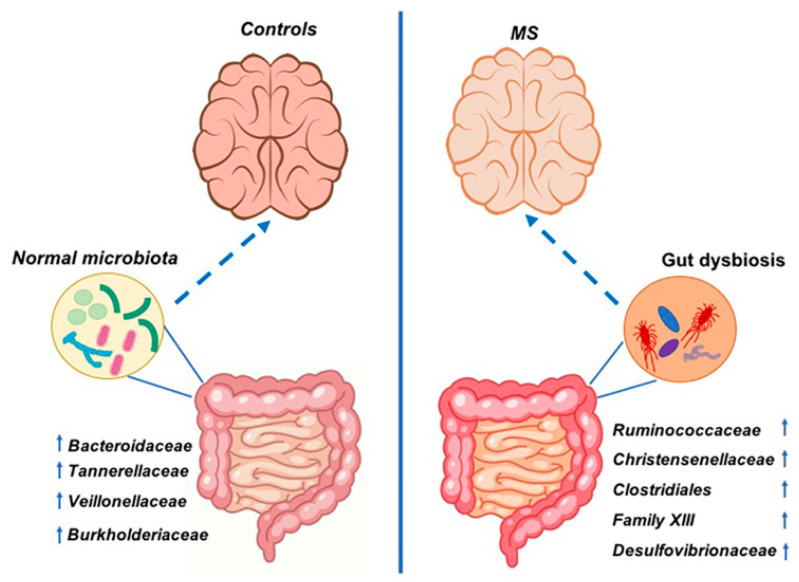
Scheme of gut bacterial variations in control subjects and MS patients coming from Sicily (Italy). Microbiota of MS patients is characterized by the increase of Ruminococcaceae, Christensenellaceae, Clostridiales vadin BB60 group, Family XIII and Desulfovibrionaceae families. Microbiota of household relatives is characterized by the increase of Bacteroidaceae, Tannerellaceae, Veillonellaceae and Burkholderiaceae families. The arrows next to the families’ names indicate that the bacterial family was found increased or decreased in this study. The discontinuous arrows represent the interaction between the gut microbiota and the brain.

This study, for the first time, investigated the composition of the gut microbiome of MS patients and their household relatives who came from a delimitated geographical location (Sicily, Italy). Analyses conducted in this study demonstrated a state of intestinal dysbiosis in patients with MS compared to controls living under the same environmental conditions. Data suggested that MS patients share microbial components, mainly when the patients have an EDSS of 4.5–7, which might be involved in exacerbations of the symptoms of the disease. In this study, there was an increase of bacterial families, such as Ruminococcaceae, Christensenellaceae, Clostridiales, Family XIII, and Desulfovibrionaceae in MS patients and a decrease of Bacteroidaceae, Veillonellaceae, Tannerellaceae, and Burkholderiaceae in respect to control subjects (Figure 8). These differences are likely not due to environmental factors, since the patients and the household relatives were asked to follow the same diet in the weeks before sample collection. These findings highlight how studying a cohort coming from the same area and sharing the same environmental conditions is important to understand the MS-associated gut microbiota.

Furthermore, our data highlight the role of environmental factors and lifestyle on the gut microbiome, since we found different microbial compositions of our samples and those reported by the Northern Italy study [9]. Knowledge of the bacterial network that promotes and maintains inflammation may in the future address specific therapeutic interventions to affect the microbiome or to identify biomarkers to assess the presence and/or progression of the disease.

Furthermore, since heterogeneous results have been obtained in different studies, it is necessary to keep in mind that any therapeutic intervention is strictly dependent upon the environmental conditions in which patients live.

## Figures and Tables

**Table 1:** Subjects of the study were grouped according to sex, age, and EDSS.

Cohort	n. Subjects	ID Family	Sex (M/F)	Age Range	EDSS
Controls	15	-	8/7	21–69	-
RRMS	9	1,**2**,6,**8**,11, 12,13,14,**15**	3/6	28–65	1–4.5
RRMS	5	4,5,7,9,10	1/4	54–66	5–7
PPMS	1	3	0/1	57	7.5

ID family is the number to identify the MS patient and the relative. 11S and 12S were collected from two naïve MS patients, and samples 2, 8 and 15 derived from relatives of the same sex (2 and 15 are mother and daughter; 8 are sisters), in which one is affected by MS.

**Table 2:** Diversity indices of the studied samples.

Sample	S	Good’s Coverage	Chao1	ACE	α	1-D	H’	e
1S	27	1.00	96.87	89.33	6.14	0.14	2.48	0.75
1C	60	0.99	99.39	90.94	5.7	0.08	3.13	0.76
2S	29	0.99	102.45	93.46	8.34	0.13	2.51	0.74
2C	20	1.00	102.76	94.44	5.65	0.14	2.32	0.77
3S	31	1.00	104.16	95.97	3.12	0.09	2.80	0.81
3C	21	0.99	106.54	97.32	10.9	0.18	2.14	0.70
4S	35	0.99	107.76	98.98	7.51	0.13	2.55	0.71
4C	39	1.00	111.43	101.82	5.79	0.10	2.78	0.76
5S	31	0.99	115.32	104.3	7.96	0.16	2.41	0.70
5C	26	1.00	117	105.95	6.53	0.10	2.61	0.80
6S	23	0.99	117.71	106.6	4.56	0.19	2.16	0.69
6C	19	1.00	117.52	107.63	5.31	0.20	2.07	0.70
7S	25	1.00	117.46	108.67	5.28	0.14	2.31	0.71
7C	32	1.00	118.21	109.54	6.40	0.14	2.50	0.72
8S	26	0.99	118.66	110.2	9.03	0.12	2.51	0.77
8C	25	1.00	119.18	111.56	5.92	0.15	2.28	0.70
9S	24	0.99	121.68	113.24	7.48	0.13	2.47	0.77
9C	29	1.00	122.43	114.51	4.51	0.07	2.86	0.85
10S	29	0.99	123.61	115.65	6.55	0.13	2.48	0.73
10C	21	1.00	126.93	118.21	6.61	0.16	2.24	0.73
11S	26	0.99	43.27	41.56	5.73	0.21	2.13	0.65
11C	20	1.00	59.09	50.84	6	0.20	2.03	0.68
12S	31	0.99	127.94	119.14	8.58	0.17	2.42	0.70
12C	35	0.99	128.72	119.86	6.82	0.13	2.61	0.73
13S	29	1.00	68.26	61.7	7.03	0.13	2.49	0.74
13C	28	1.00	73.39	69.46	6.42	0.17	2.28	0.68
14S	29	1.00	80.15	74.66	8.31	0.20	2.21	0.65
14C	21	0.99	82.23	78.6	3.85	0.17	2.17	0.71
15S	32	0.99	86.94	84.29	8.81	0.12	2.59	0.74
15C	29	1.00	91.01	85.36	7	0.14	2.45	0.72

S: total number of families; Chao1 and ACE: abundance-based richness estimators; α: alpha diversity; 1-D: Simpson’s index; H’: Shannon–Wiener diversity; e: evenness. White and grey lines indicate MS patients’ and controls’ indices, respectively. Patients are indicated as number-S, controls as number-C.

## Data Availability

Sequences were deposited in GenBank (BioProject ID: PRJNA684124) and will be available after acceptance for publication of the manuscript.

## Supplementary Materials

The following are available online at https://www.mdpi.com/article/10.3390/life11070620/s1, Table S1: Relative abundance (%) of family-specific 16S rRNA gene amplicon sequences in EDSS 1–4.5 group, Table S2: Relative abundance (%) of family-specific 16S rRNA gene amplicon sequences in EDSS 5–7 group. Figure S1: Metabolic activities of microbial community present in MS patients with EDSS 1–4.5 and controls, Figure S2: Metabolic activities of microbial community present in MS patients with EDSS 5–7 and controls.
