# Supplementary material for: Comparison of the Intestinal Microbiome of Italian Patients with Multiple Sclerosis and Their Household Relatives
Source: Life (Basel). 2021 Jun 26;11(7):620. doi: 10.3390/life11070620 (PMC8307959; doi:10.3390/life11070620)
Supplement: Supplementary file 1 [file life-11-00620-s001.zip › life-1248362-supplementary.pdf]

life

MDPI

Article

Comparison of the Intestinal Microbiome of Italian Patients with Multiple Sclerosis and Their Household Relatives

Paola Galluzzo [1,2], Fanny Claire Capri [1], Luca Vecchioni [1], Sabrina Realmuto [3], Luca Scalisi [4], Salvatore Cottone [5], Domenico Nuzzo [6,*] and Rosa Alduina [1,*]

1 Dipartimento Scienze e Tecnologie Biologiche, Chimiche e Farmaceutiche, Viale delle Scienze, University of Palermo, 90133 Palermo, Italy; paola.galluzzo@unipa.it (P.G.); fannyclaire.capri@unipa.it (F.C.C.); luca.vecchioni@unipa.it (L.V.)
2 Istituto Zooprofilattico Sperimentale della Sicilia "A. Mirri", via G. Marinuzzi 3, 90129 Palermo, Italy
3 Centro Sclerosi Multipla, UOC Neurologia e Stroke Unit, AOOR Villa Sofia Cervello, 90146 Palermo, Italy; sabrina.realmuto@pec.it
4 Centro Medico di Fisioterapia "Villa Sarina", Via Porta Palermo 123, 91011 Alcamo, Italy; lucasfrog@libero.it
5 U.O.C. Neurologia con Stroke Unit A.R.N.A.S. Civico, 90127 Palermo, Italy; salvatore.cottone1@arnascivico.it
6 Istituto per la Ricerca e l'Innovazione Biomedica, CNR, Via U. La Malfa 153, 90146 Palermo, Italy; domenico.nuzzo@irib.cnr.it
* Correspondence: domenico.nuzzo@irib.cnr.it (D.N.); valeria.alduina@unipa.it (R.A.); Tel.: +39 091-23897306 (R.A.)

Abstract: Multiple sclerosis (MS) is a chronic immune-mediated disease of the central nervous system, caused by a combination of genetic and environmental factors. In recent years, a role in MS pathogenesis was assigned to the gut microbiota. However, different signatures of gut dysbiosis have been shown to depend on environmental factors, like diet and lifestyle. In this study, we compared the gut microbiome in MS patients and their household healthy relatives sharing lifestyle and environmental factors. Faecal metagenomic DNA was extracted and the V3–V4 regions of the conserved bacterial 16S ribosomal RNA gene were amplified and sequenced. While overall bacterial communities were similar, specific families differed between healthy and MS subjects. We observed an increase in Ruminococcaceae, Christensenellaceae, Desulfovibrionaceae, Clostridiales, and Family XIII in MS patients, while Bacteroidaceae, Tannerellaceae, Veillonellaceae, and Burkholderiaceae were more abundant in healthy controls. In addition, principle coordinate analysis showed that the gut microbiome of all MS patients formed a cluster being less diverse than the household relatives and that gut microbiota of MS patients with EDSS 4.5–7 formed a distinct cluster in respect to their controls. Overall, our study is consistent with the hypothesis that MS patients have gut microbial dysbiosis and evidenced the importance of environmental factors in shaping the gut microbiome.

Keywords: multiple sclerosis; microbiome; Ruminococcaceae; Desulfovibrionaceae Christensenellaceae; Clostridiales; Bacteroidaceae; Tannerellaceae; Veillonellaceae; Burkholderiaceae

Citation: Galluzzo, P.; Capri, F.C.; Vecchioni, L.; Realmuto, S.; Scalisi, L.; Cottone, S.; Nuzzo, D.; Alduina, R. Comparison of the Intestinal Microbiome of Italian Patients with Multiple Sclerosis and Their Household Relatives. *Life* **2021**, *11*, 620. https://doi.org/10.3390/life11070620

Academic Editors: Fabiana Piscitelli and Tiziana Bisogno

Received: 20 May 2021
Accepted: 23 June 2021
Published: 26 June 2021

Publisher's Note: MDPI stays neutral with regard to jurisdictional claims in published maps and institutional affiliations.

Copyright: © 2021 by the authors. Submitted for possible open access publication under the terms and conditions of the Creative Commons Attribution (CC BY) license (http://creativecommons.org/licenses/by/4.0/).

Supplementary Materials:

Table S1. Relative abundance (%) of family-specific 16S rRNA gene amplicon sequences in EDSS 1-4.5 group. The rows with statistically significant ($p \leq 0.05$) are indicated with *.

Family	MS	C	*p-value*
Ruminococcaceae	30.55	23.65	0.019*
Bacteroidaceae	7.93	12.96	0.004*
Burkholderiaceae	2.45	3.16	0.403
Prevotellaceae	3.43	1.35	0.093
Christensenellaceae	3.52	1.08	0.004*
Clostridiales vadinBB60 group	2.45	1.48	0.141
Tannerellaceae	2.22	1.60	0.164
Veillonellaceae	1.52	1.90	0.312
Desulfovibrionaceae	1.66	0.85	0.007*
Family XIII	1.02	0.87	0.647
Akkermansiaceae	0.40	0.67	0.350

Table S2. Relative abundance (%) of family-specific 16S rRNA gene amplicon sequences in EDSS 5-7 group. The rows with statistically significant ($p \leq 0.05$) are indicated with *.

Family	MS	C	*p-value*
Ruminococcaceae	28.83	20.45	0.020*
Bacteroidaceae	9.06	11.78	0.198
Burkholderiaceae	1.63	3.44	0.007*
Prevotellaceae	3.09	3.42	0.872
Christensenellaceae	3.58	1.13	0.014*
Clostridiales vadinBB60 group	3.31	0.44	0.003*
Tannerellaceae	1.74	3.31	0.026*
Veillonellaceae	1.21	2.81	0.012*
Desulfovibrionaceae	1.17	0.85	0.458
Family XIII	1.17	0.42	0.019*
Akkermansiaceae	0.28	0.00	0.042*

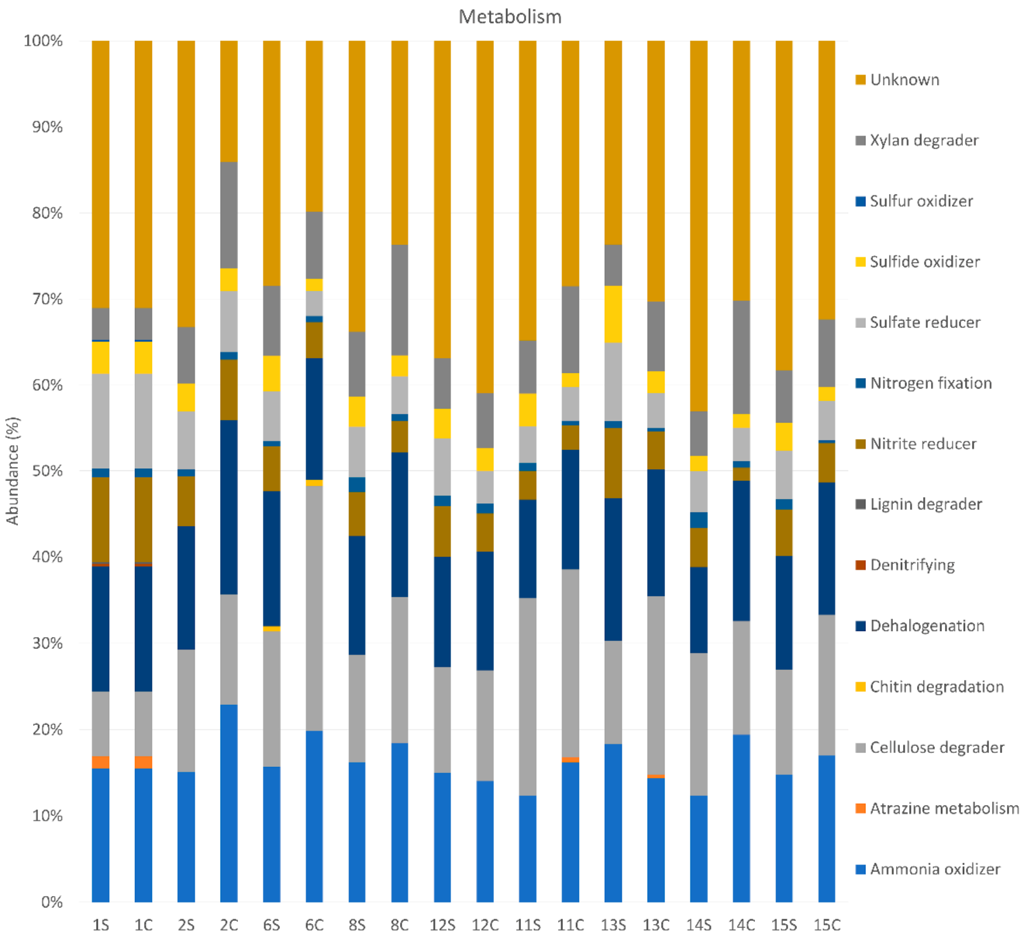

Figure S1. Metabolic activities of microbial community present in MS patients with EDSS 1–4.5 and controls.

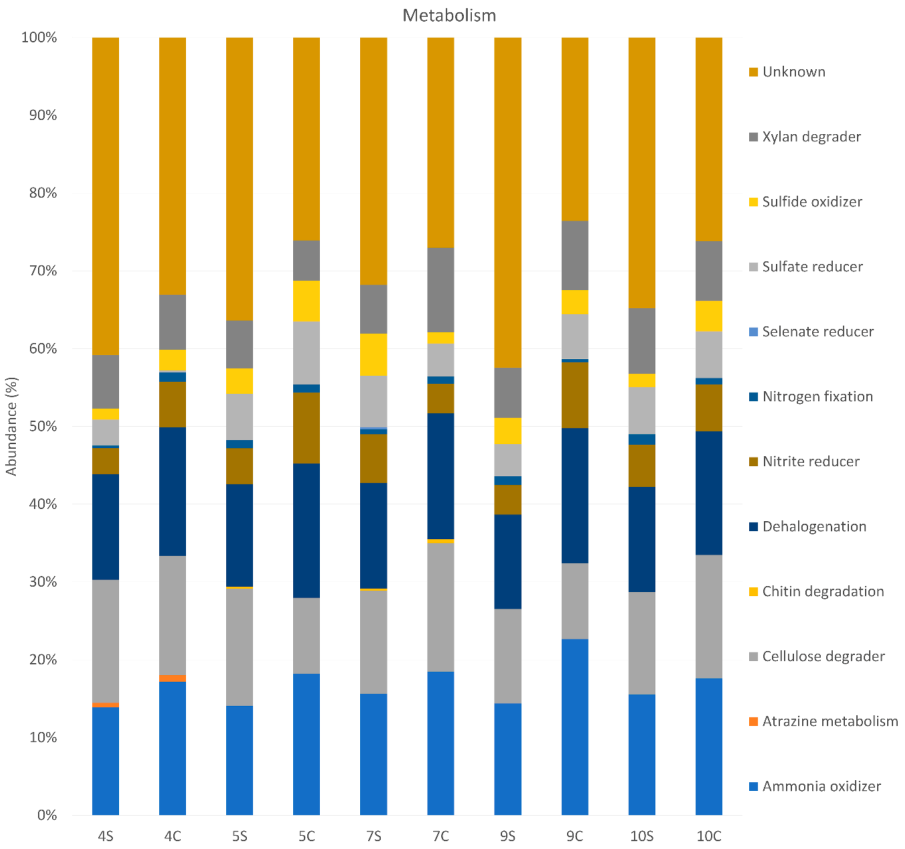

Figure S2. Metabolic activities of microbial community present in MS patients with EDSS 5–7 and controls.
